# Supplementary material for: A Systematic Review of the Association of Skipping Breakfast with Weight and Cardiometabolic Risk Factors in Children and Adolescents. What Should We Better Investigate in the Future?
Source: Nutrients. 2019 Feb 13;11(2):387. doi: 10.3390/nu11020387 (PMC6412508; doi:10.3390/nu11020387)
Supplement: Supplementary file 1 [file nutrients-11-00387-s001.pdf]

Supplementary Table 1. Newcastle - Ottawa Quality Assessment Scale for studies on weight and skipping breakfast

Reference (Author, year, n)	Selection	Comparability	Outcome/ Exposure
Dialektakou 2008 [12]	***	**	*
Dubois 2008 [13]	***	*	**
Harding 2008 [14]	****	*	*
Duncan 2008 [15]	***	*	**
Croezen 2009 [16]	***	**	**
Nagel 2009 [17]	***	*	*
Sun 2009 [18]	***	**	*
Maddah 2010 [19]	***	*	*
Isacco 2010 [20]	***	*	*
Deshmukh-Taskar 2010 [21]	***	*	*
So 2011 [22]	***	*	**
Tin 2011 [23]	***	*	*
Mushtaq 2011 [24]	****	**	**
Kuriyan 2012 [25]	***	*	*
Kyriazis 2012 [26]	***	*	*
Van Lippevelde 2013 [27]	***	*	*
Januszek-Trzciąkowska 2014 [28]	***	*	*
Kupers 2014 [29]	***	*	**
O'Neil 2015 [30]	***	*	**
Smetanina 2015 [31]	***	*	**
Zakrzewski 2015 [32]	***	*	**
Wijtzes 2016 [33]	***	*	**
Fayet-Moore 2016 [34]	***	*	*
Alsharairi 2016 [35]	***	*	*
Fayet-Moore 2017 [36]	***	*	*
Smith	***	*	*

2017 [37]			
Gotthelf 2017 [38]	***	*	**
Nilsen 2017 [39]	***	*	**
Kesztyus 2017 [40]	****	*	**
Zalewska 2017 [41]	***	*	*
Coulthard 2018 [42]	***	*	**
Tee 2018 [43]	***	*	**
Archero 2018 [44]	***	*	*
Smith 2010 [11]	***	*	*
Shafiee 2013 [45]	***	*	**
Ho 2015 [46]	***	*	**
Marlatt 2016 [47]	***	*	**

Supplementary Table 2. Newcastle - Ottawa Quality Assessment Scale for studies on metabolic variables and skipping breakfast

**Reference (Author, year, n)**	**Selection**	**Comparability**	**Outcome/ Exposure**
Smith 2010 [11]	***	*	*
Monzani 2013 [48]	***	*	*
Shafiee 2013 [45]	****	*	**
Ho 2015 [46]	****	*	**
Osawa 2015 [49]	***	*	**
Marlatt 2016 [47]	****	*	**
